# Effect of Fat Type and Mango Peel Powder on the Physicochemical Properties of Beef Patties During Cold Storage and In Vitro Digestion

**DOI:** 10.1155/2024/2981134

**Published:** 2024-10-23

**Authors:** Martha Olivia Vázquez-Meza, Humberto González-Ríos, Gustavo Adolfo González-Aguilar, Manuel Viuda-Martos, José Luis Dávila-Ramírez, Martín Valenzuela-Melendres

**Affiliations:** ^1^Research Center for Food and Development, CIAD Carretera Gustavo Enrique Astiazarán Rosas, No. 46, Hermosillo, Sonora 83304, Mexico; ^2^IPOA Research Group, Agro-Food and Agro-Environmental Research and Innovation Center, Miguel Hernández University (CIAGRO-UMH), Ctra. Beniel km 3.2, 03312 Orihuela, Alicante, Spain

**Keywords:** beef patties, in vitro digestion, lipid oxidation, mango peel

## Abstract

The aim of this research was to evaluate the effects of fat type and mango peel powder (MP) on the physicochemical properties of cooked beef patties during cold storage and after in vitro digestion. Beef patties were prepared with saturated beef fat (BF) and pre-emulsified avocado oil (AO) or pre-emulsified safflower oil (SO). MP was added at 0% or 1%. The treatments were as follows: T1 (BF, no added MP), T2 (AO, no added MP), T3 (SO, no added MP), T4 (BF + 1%MP), T5 (AO + 1%MP), and T6 (SO + 1%MP). Substituting saturated fat with AO and SO improved the fatty acid profile of beef patties. The addition of pre-emulsified oils increased (*p* < 0.05) the *L*^∗^, *a*^∗^, and *b*^∗^ values. Moreover, the incorporation of MP in the meat formulation decreased (*p* < 0.05) lipid oxidation during cold storage. Adding MP to the meat formulation decreased (*p* < 0.05) lipid oxidation before and after in vitro digestion. Replacement of saturated fat with vegetable oils and incorporation of MP may be an alternative strategy to improve the quality of beef patties during cold storage and decrease lipid oxidation after in vitro digestion.

## 1. Introduction

The demand for ready-to-eat (RTE) foods has increased, and their popularity could be attributed mainly to changes in sociodemographic characteristics and consumer lifestyles [1]. One of the most consumed RTE products is beef patties, which are inexpensive and tasty [2]. During the preparation of beef patties, they are subjected to processes such as grinding, preheating, storage, and heating before eating, which leads to enhanced lipid oxidation and decreased product quality if reheating occurs [1]. Beef patties are widely consumed due to their characteristic sensory properties of flavor and aroma and their high-quality protein, B complex vitamins, and minerals such as iron and zinc [3]. However, these products are also a source of saturated fats, cholesterol, and salt, which are considered risk factors for cardiovascular disease [4]. In this sense, society has exhibited changes in eating habits, and people are increasingly interested in low-fat products and food that has been nutritionally improved [5]. An alternative for consumers looking for healthy foods is to replace saturated fats with vegetable oils during food manufacturing.

Vegetable oils are rich sources of monounsaturated fatty acids (MUFAs) and polyunsaturated fatty acids (PUFAs), which are considered more beneficial for human health compared to beef fat (BF) [6]. However, unsaturated fatty acids have double bonds, making them more prone to oxidation when exposed to pro-oxidant conditions [7]. Despite this, there is a greater preference for consuming oils rich in oleic acid, such as avocado oil (AO) and safflower oil (SO). These oils have a beneficial effect on human health and are an essential source of compounds such as tocopherols and phytosterols [8].

Carvajal-Zarrabal et al. [9] reported that the lipid content in AO, mainly MUFAs, is associated with cardiovascular system benefits and anti-inflammatory effects. Researchers are investigating the creation of AO–based products. Arancibia et al. [10] proposed the use of nanoemulsions with AO to increase the dispersibility of water in oils, improve its physical and chemical stability, and enhance the bioavailability of the oil's components. In this way, the potential health benefits of the oil can be fully utilized. Incorporating AO into the diet of healthy adults has been found to have a positive impact on postprandial insulin and blood glucose levels, as well as on key markers of cardiovascular health such as total cholesterol, low-density lipoproteins, and triglycerides. Additionally, the use of AO has been shown to help reduce inflammation, as evidenced by decreases in the levels of C-reactive protein and interleukin-6. These findings highlight the potential health benefits of including AO in the diet [11]. SO has a high content of linoleic acid, which can improve lipid metabolism and reduce blood cholesterol levels [12], as well as protect against fractures and bone loss [13]. These findings on the health-promoting potential of AO and SOs indicate the importance of developing widely consumed foods enriched with these types of oils.

Lipids are prone to oxidation during food preparation, cooking, and storage, which results in the formation of primary products, such as malondialdehyde (MDA), 4-hydroxy-nonenal (4-HNE), and 4-hydroxy-hexenal (4-HHE). Even after food is consumed, lipid oxidation can increase when it is exposed to pro-oxidant conditions in the gastrointestinal tract, including low pH levels and metal ions in the food bolus [14]. The products of lipid oxidation can cause oxidative damage to biological systems, as shown in studies conducted by Liao, Zhu, and Chen [15] and Requena et al. [16]. For these reasons, it is necessary to look for alternatives to delay lipid oxidation in meat products during their shelf life and digestion. A natural option is to use by-products with bioactive compounds, such as mango peel (MP). Mango processing generates approximately 3 million tons of seeds and peels annually, representing between 25% and 40% of the fruit [17]. Asian countries are the leading producers of mango, with 77% of production, while American and African countries produce 13% and 9% of the total, respectively [18]. In addition to the availability of mango by-products, this ingredient has attracted interest because it contains bioactive compounds such as polyphenols, carotenes, vitamins, and mangiferin [19–21]. These compounds could exert health benefits or improve the quality of a meat product if it is incorporated into the formulation.

MP also contains a considerable amount of dietary fiber [22, 23]. This fiber is resistant to hydrolysis by endogenous enzymes present in the small intestine [24]. According to Abbasi et al. [25] and Tokas et al. [26], MP can be a beneficial addition to food products due to its fiber content, suggesting that it can enhance the functional properties of foods.

MP also has anti-inflammatory, antitumor, and antioxidant activities attributed to the mangiferin (C-glucosylxanthone) present in the peel [21, 27]. In addition, the presence of carotenoids in MPs can reduce the risk of developing cancer, cardiovascular diseases, and bone disorders [28].

Studies have been carried out to evaluate the reduction of lipid oxidation in meat products by the addition of natural antioxidants [29]. However, these types of studies have focused on cold storage quality rather than the digestion process. There are few investigations reported in the literature that include both shelf life and in vitro digestion studies of meat products. Torres-Martínez et al. [30] evaluated the effects of *Pleurotus ostreatus* powder on the oxidative stability of pork patties during cold storage and after in vitro gastrointestinal digestion. The authors found that *P. ostreatus* enhances meat quality traits in refrigerated samples and increases their phytochemical content and antioxidant activity after in vitro digestion. Biasi et al. [31] studied the effect of goldenberry flour on the quality of bologna-type mortadella during cold storage and after in vitro digestion. The authors reported that goldenberry flour affected important quality characteristics of the product, including pH, texture, and color, and increased the antioxidant activity and phenolic compound content after in vitro digestion.

According to the reviewed literature, limited research exists on the use of MP as a natural antioxidant in meat products. The studies available only focus on the shelf life of the product, and there is no research available on in vitro digestion. Furthermore, additional research is needed on meat products prone to oxidation, especially those in which saturated fats are substituted with vegetable oils such as safflower and avocado. Research on the use of AO and SO as replacements for saturated fats in meat products, such as beef patties, and their effects on quality during refrigerated storage and after in vitro digestion, as well as the use of MP as a natural antioxidant, can provide valuable insights to the food industry, the scientific community, and health-conscious consumers. Therefore, the objective of the present investigation was to evaluate the effects of the addition of MP and fat type to cooked beef patties on the physicochemical quality during cold storage and the reduction in lipid oxidation after in vitro digestion.

## 2. Materials and Methods

### 2.1. Materials

Beef (semimembranosus m., 48 h postmortem), bovine back fat, AO (Olew, Mexico), and SO (Oleico, Mexico) were purchased from a local market in Hermosillo, Sonora, Mexico. Pre-emulsions were prepared by emulsifying vegetable oils (45%) with soy protein (10%) and water (45%). The emulsions were stored at 4°C prior to beef patty manufacture. MP was obtained from commercially ripe mangoes in the local market. The fruit was washed with soap and water, disinfected with 0.1% sodium hypochlorite, and peeled with a manual peeler. The MPs were frozen at −35°C and then freeze-dried in a tray freeze-dryer (FreeZone 6 Liter Benchtop, Labconco Corporation, Kansas City, United States) for 72 h. The freeze-dried MPs were ground in a mill until a powder with a particle size of approximately 0.1 mm was obtained.

### 2.2. Beef Patty Preparation

The beef was cut from all exterior and connective tissue and minced (Model 4152, Hobart Dayton, OH, United States) through the opening of a hole plate of 4.5 mm. Raw beef patties were prepared by hand mixing ground meat with saturated BF, pre-emulsified AO, or SO oils at levels of 20% in the formulation. MP powder was added at 0 or 1%. Beef patties with a weight of 60 g, diameter of 80 mm, and thickness of 10 mm were manually formed. Six batches were prepared, which resulted in the following treatments: T1 (BF without added MP), T2 (AO without added MP), T3 (SO without added MP), T4 (BF + 1%MP), T5 (AO + 1%MP), and T6 (SO + 1%MP). The raw beef patties were cooked with a grill until they reached an internal temperature of 71.1°C. The samples were cooled to room temperature (25°C), individually placed on Styrofoam plates, packaged with polyvinyl chloride film, and stored for 1, 4, or 8 days at 4°C. The total number of beef patties prepared was 108: three patties for each treatment, six treatments (T1, T2, T3, T4, T5, and T6), three storage times (1, 4, and 8 days), and two independent replications.

### 2.3. Physicochemical Properties of Beef Patties

#### 2.3.1. Chemical Composition

The moisture, fat, protein, and ash contents were determined in triplicate using established AOAC (2000) methods. Moisture determination was performed using a drying oven at a temperature of 100°C for 16 h (Method 950.46). The ash content was determined in a muffle furnace at 550°C for 5 h (Method 920.153). Fat was quantified by the Goldfish extraction method from dry samples (Method 960.39). Finally, the protein content of the dry and defatted samples was determined via the Microkjeldahl method (Method 981.10).

#### 2.3.2. Fatty Acid Profile

Lipid extraction from beef patties was performed using chloroform:methanol (2:1 v/v) following the procedure reported by Bligh and Dyer [32]. After extraction, the solvent was removed from the lipid extracts in a 35°C water bath under a nitrogen atmosphere before fatty acid methylation. The transmethylation of lipid extracts was carried out in the presence of boron trichloride according to the method of Park and Goins [33].

To determine the composition of fatty acid methyl esters (FAME), an Agilent gas chromatograph (Model 7890 B, Santa Clara, CA, United States) equipped with an autosampler (Model 7693) and a flame ionization detector (FID) was used. The fatty acids were separated on a fused silica capillary column (SP-2560, Supelco, Bellefonte, PA, United States) with an internal diameter of 0.25 mm and a length of 100 m. The oven temperature was programmed from an initial temperature of 150°C (20 min) to a final temperature of 220°C at a speed of 5°C/min. The injector temperature was set at 250°C, and the FID temperature was 300°C. The carrier gas used was 17 psi of hydrogen, and nitrogen was used as the filler gas. ChemStation software (ChemStation, Agilent Santa Clara, CA, United States) was used to integrate the chromatograms. Fatty acids were identified by comparing retention times with those of commercial standards (Supelco 37 component FAME Mix, Bellefonte, PA, United States) and are expressed as a percentage of total fatty acids. The total percentages of saturated fatty acids (SFAs), MUFAs, and PUFAs were calculated.

#### 2.3.3. Instrumental Color (CIE *L*^∗^, *a*^∗^, and *b*^∗^) and pH

Instrument color was evaluated on the surface of each sample. A colorimeter (Chroma meter CR-400, Konica Minolta Sensing, Inc., Osaka, Japan) was used. The color measurement included the determination of the *L*^∗^ (lightness), *a*^∗^ (redness), and *b*^∗^ (yellowness) values. Five determinations per sample were made, and the average per treatment was reported. pH measurements were performed directly on the samples using an HI98140 puncture potentiometer (Hanna, Woonsocket, RI, United States).

#### 2.3.4. Lipid Oxidation

The amount of malondialdehyde (MDA) was measured in the treatments during Days 1, 4, and 8 of storage at 4°C by using the thiobarbituric acid reactive substances methodology described by Pfalzgraf, Frigg, and Steinhart [34]. For this assay, 5 g of sample was mixed with 15 mL of trichloroacetic acid and homogenized for 1 min at 11,000 rpm. Subsequently, the homogenized sample was centrifuged at 2300 × g for 30 min at 5°C. After the supernatant was filtered (Whatman filter paper No. 1), 2 mL of the filtrate was mixed with 2 mL of thiobarbituric acid. The resulting mixture was homogenized for 30 s and then placed in a water bath at 99°C for 40 min. Afterward, the tubes were cooled, and the absorbance at 532 nm was measured using a UV–Vis spectrophotometer (Cary-60 UV–Vis spectrophotometer, Agilent Technologies, Santa Clara, CA, United States). Lipid oxidation was calculated based on the TBA content using a standard curve of 1,1,3,3-tetramethoxypropane and expressed as a milligram of MDA/kg of sample.

### 2.4. In Vitro Digestion Analysis

In vitro digestion was performed following the methodology described by Brodkorb et al. [35] with some modifications. Seven grams of sample was weighed, mixed with 11 mL of simulated salivary fluid, and homogenized in an ultraturrax at 11,000 rpm; subsequently, 150 U/mL of porcine *α*-amylase was added, mixed with CaCl_2_ (0.15 mM), and immersed in a shaking bath for 2 min at 37°C. After that, 11 mL of simulated gastric fluid containing 2000 U/mL of porcine pepsin was added and combined with gastric lipase (60 U/mL) and CaCl_2_ (0.15 mM). The pH of the digest was adjusted to 3.0 with 6 M HCl. Then, the gastric bolus was incubated for 2 h at 37°C in a shaking bath. To simulate the intestinal phase of digestion, 11 mL of 100 U/mL pancreatic fluid, 5 mL of bile fluid (10 mM), and CaCl_2_ (0.6 mM) were added, and the pH was adjusted to 7 before incubation for 2 h at 37°C in a shaking bath. Once the time had elapsed, the samples were frozen at −20°C to stop the enzymatic reaction and stored for further analysis. Free fatty acids (FAAs) were measured at the end of the in vitro digestion, while total phenol, antioxidant capacity, and lipid oxidation were measured before and after in vitro digestion.

#### 2.4.1. FAA

The determination of FAAs was performed according to the methodology of Zhou et al. [36] with some modifications. Initially, 0.1 g of the sample was weighed and mixed with 2 mL of distilled water. The mixture was then vortexed for 45 s. After that, 5 mL of chloroform:methanol (*v*/*v* = 2/1) was added to extract the FAAs, followed by centrifugation at 4000 × g for 3 min at 15°C. The lower phase was collected in a 300 mL flask, to which 100 mL of ether/ethanol (1:1 ratio) was added. Approximately 2–3 drops of phenolphthalein were added to the mixture for subsequent titration with 0.01 M NaOH until the color of the sample changed from colorless to light pink. The milligram of FFAs released per gram of meat was calculated using the following equation:

 $$\begin{array}{c} \text{FFA }\left(\frac{\text{mg}}{\text{g}}\right)=\frac{\left(V_{\text{NaOH}}*C_{\text{NaOH}}*M_{\text{FFA}}\right)}{W_{\text{sample}}} \end{array}$$

where *V*_NaOH_ is the concentration of the NaOH solution (0.01 M), *M*_FFA_ is the molecular weight of the FFAs in the oil phase (280 g/mol), and *W*_sample_ is the weight of the sample (g).

#### 2.4.2. Phenolic Compound Extraction

Phenolic compound extraction was performed following the methodology described by Sogi et al. [37] with some modifications. First, 20 mL of 80% ethanol was mixed with 1 g of sample and homogenized for 30 s at 11,000 rpm (Ultra Turrax X25, IKAWerke, United States). Subsequently, the mixture was sonicated (Branson, 2510R-DTH) for 30 min and centrifuged at 9,300 rpm for 15 min at 4°C. The supernatant was filtered (Whatman filter paper No. 1), and the extraction was repeated twice, adding 20 mL of the solvent for each wash. The supernatant was diluted to 50 mL with 80% ethanol. Finally, the extracts were stored at –20°C until they were used to measure the total phenol content (TPC) and antioxidant capacity (2,2⁣′-azino-bis-(3-ethylbenzothiazoline-6-sulfonic acid, ABTS and ferric reducing antioxidant power, ferric-reducing power (FRAP) assays).

#### 2.4.3. TPC and Antioxidant Capacity

The TPC was determined by the method described by Singleton and Rossi [38] with some modifications. Briefly, 30 *μ*L of the extract was mixed with 150 *μ*L of Folin–Ciocalteu's reagent. After 8 min of equilibration, 120 *μ*L of Na_2_CO_3_ (7.5%) was added, and the mixture was incubated for 30 min in the dark. The absorbance at 765 nm was then measured on a Flourstar Omega microplate reader (BMG Labtech Inc., Durham, NC, United States). The concentration of total phenols was calculated using a standard curve of gallic acid, and the results are expressed as mg of gallic acid equivalents (GAE)/g dry weight.

Inhibition of the ABTS+ radical (2,2-azinobis-[3-ethylbenzothiazoline-6-sulfonic acid) was performed according to the methodology of Re et al. [39]. The ABTS+ radical cation was produced by reacting an ABTS stock solution (7 mM) with K_2_S_2_O_8_ (0.139 mM) in the dark at 25°C for 14–16 h before use. Subsequently, the absorbance was adjusted to 0.7 ± 0.02 by dilution in 95% ethanol. After that, 295 *μ*L of ABTS solution was mixed with 5 *μ*L of methanolic sample in a microplate. The absorbance was read after 5 min of initial mixing at 734 nm in a Flourstar Omega microplate reader (BMG Labtech Inc., Durham, NC, United States). The results were calculated using a Trolox standard curve and expressed as mg of Trolox equivalents (TE)/g dry weight.

The FRAP assay was performed by mixing 0.5 mL of TPTZ (2,4,6-tris(2-pyridyl)-s-triazine), 0.5 mL of acetate buffer, 0.5 mL of FeCl_3_, and 20 *μ*L of sample with 290 *μ*L of FRAP reagent in a 96-well microplate. The reaction mixture was incubated at 37°C for 15 min. The absorbance at 595 nm was measured on a Flourstar Omega microplate reader (BMG Labtech Inc., Durham, NC, United States). The Trolox reagent was dissolved in ethanol to establish the standard calibration curve.

### 2.5. Statistical Analysis

The statistical data analysis was performed using Number Cruncher Statistical Systems 2022 (NCSS, Kaysville, UT) software. The experiment consisted of six treatment groups (T1, T2, T3, T4, T5, and T6) and three storage times (1, 4, and 8 days) with two replications. In each replicate, all response variables were measured in triplicate. The chemical composition and fatty acid profile were evaluated on Day 1, while FAA content was measured after the in vitro digestion. The effect of the treatments on these three response variables was analyzed by one-way analysis. Two-way ANOVA was used to determine the effect of treatment and storage conditions and their interaction on the quality parameters of the beef patties (instrument color, pH, lipid oxidation, TPC, and antioxidant capacity). Treatments and storage days were assigned as fixed effects, and replications were assigned as random effects. When significant differences between treatments were found, the Tukey–Kramer test was performed (*p* < 0.05).

## 3. Results and Discussion

### 3.1. Physicochemical Properties of Beef Patties

#### 3.1.1. Chemical Composition

Table 1 shows the results of the chemical composition of cooked beef patties supplemented with different types of fat and MP. No differences (*p* > 0.05) were found in the proximal composition of the evaluated treatments since they presented similar moisture (53.36%–55.72%), fat (11.49%–13.66%), protein (25.01%–27.51%), and ash (4.45%–5.19%) contents. The above results are presented from the lowest to the highest values. The treatments evaluated in this study were designed with equal fat quantities, with 20% being saturated BF (T1 and T4), 20% being pre-emulsified AO (T2 and T5), and 20% being pre-emulsified SO (T3 and T6). The key point of interest was the similar fat content in these treatments, leading to the expectation that the moisture, protein, fat, and ash content in the beef patties would remain unchanged. These results are in agreement with those of Yüncü et al. [40], who reported moisture (57.32%), fat (13.21%), protein (25.86%), and ash (4.02%) contents in beef patties cooked by the panfrying method. In addition, Rodríguez-Carpena, Morcuende, and Estévez [41] reported similar results in cooked pork hamburgers made with avocado, sunflower, and olive oils as substitutes for pork fat. All treatments evaluated in our study had high protein and low-fat content. The protein content was greater than that of beef patties marketed in Mexico, as reported by the Federal Consumer Attorney's Office (2019), for which the protein content ranges from 10.2% to 14.6%. However, in American products, the USDA reported protein values of 25.8%, similar to the results of the present study [42]. Adding different fat types and MP powder did not modify the basal chemical composition of the meat product.

#### 3.1.2. Fatty Acid Profile

The fatty acid composition of bovine back fat, commercial AO, and commercial SO used in our study for the preparation of beef patties are presented in Table 2. Bovine back fat primarily contains palmitic (C16:0), stearic (C18:0), and oleic (C18:1) acids, accounting for 22.1%, 21.03%, and 45.35% of the composition, respectively. The total SFA, MUFA, and PUFA in bovine back fat were 49.31%, 48.21%, and 2.48%, respectively, which is consistent with those reported in the USDA food database [43]. AO showed a proportion of SFA, MUFA, and PUFA of 11.2%, 51.58%, and 37.28%, respectively. The SFA, MUFA, and PUFA proportions in SO resulted in values of 8.76%, 75.48%, and 15.45%, respectively. According to the literature, AO is rich in oleic acid, ranging from 49.6% to 74.2%, while SO is rich in linoleic acid, ranging from 54.3% to 77.0% [44, 45]. Our results show a significant proportion of MUFA in SO compared to AO (Table 2), which could contradict some literature; but in Mexico, varieties of safflower high in oleic acid have been developed and used to produce commercial SO [46]. Also, it is important to note that we used commercial oils with profiles similar to those reported by the USDA database for commercial SO [47] and by Wang et al. for commercial AO [48].

Table 3 shows the fatty acid profile and includes the total SFA, MUFA, and PUFA of cooked beef patties substituted with different fat types and supplemented with MP powder. The study revealed that substituting pre-emulsified AO and SOs in beef patties led to a reduction in SFAs and an increase in MUFAs and PUFAs (T2, T3, T5, and T6). As expected, the total SFAs were greater (*p* < 0.05) in beef patties with added saturated fat (T1 and T4) than in those with pre-emulsified oils. This increase could be due to the high content of SFAs, specifically palmitic acid (C16:0) and stearic acid (C18:0) in BF, as shown in Table 2. At the same time, commercial AO and SOs were rich sources of MUFAs, mainly oleic acid (C18:1 cis). Particularly, the commercial vegetable oils used in our study had an oleic content of 50.02% and 75.48% for AO and SOs, respectively (Table 2). Carvalho Barros et al. [49] reported similar results in a study in which saturated fat was replaced by tiger nut oil in beef patties. Rodríguez-Carpena, Morcuende, and Estévez [41] reported a decrease in SFAs from 36.9% in a control treatment to 25.3% in pork patties, where 50% of saturated fat was replaced with AO. Additionally, these authors observed an increase in MUFAs from 47.0% in the control treatment to 55.8% in the avocado treatment. Kaynakci and Kiliҫ [50] reported that a similar effect was observed in the meat formulation when SO was used, as demonstrated in their study on the Wiener formulation. According to these authors, substituting BF with SO resulted in a decrease in SFAs from 40.4% to 25.7% and an increase in PUFAs from 23.7% to 44.7%.

Concerning the PUFA content, in our study, the values increased (*p* < 0.05) from 6.11% in T1 (saturated fat) to 22.41% in T2 (pre-emulsified AO), and this increase was less evident (13%) in the T3 and T6 treatments with pre-emulsified SO. The commercial AO and SO used in this study are rich sources of MUFA and PUFA (Table 2). The increase in PUFA level could be attributed to the high content of linoleic acid (C18:2 cis) and linolenic acid (C18:3) found in AO and SO, as shown in Table 2, and also reported by some researchers [44, 47].

Oleic acid is an essential fatty acid found in AO and SO, and according to Rehman et al. [51], consuming this fatty acid improves the health of consumers. Oleic acid regulates factors that cause insulin resistance and type 2 diabetes mellitus. Therefore, its consumption could be an excellent strategy for preventing and treating this type of disease.

Consumer preferences have focused on foods with better nutritional profiles; in this sense, replacing saturated fat with vegetable oils in beef patties could be a good option for people looking for healthy products. However, even though one of the main strategies in the development of healthy meat products is to replace saturated fat with unsaturated oils, its incorporation into a meat formulation could increase lipid oxidation and decrease the final quality of the product. Hence, it is important to include antioxidant compounds to prevent quality loss.

#### 3.1.3. Instrumental Color and pH

Table 4 shows the instrumental color values (*L*^∗^, *a*^∗^, and *b*^∗^) measured for cooked beef patties with different types of fat and MP. The treatments with MP had lower *L*^∗^ values (*p* < 0.05) than those without MP. This lower value is because MP is a dry ingredient that can trap an important amount of water, preventing its emigration and refraction on the food surface. Furthermore, the carbohydrates contained in MP can react with meat protein by the Maillard reaction when exposed to high temperatures, forming dark pigments and decreasing the *L*^∗^ value. Similar results were reported by Manzoor, Ahmad, and Yousuf [52] for chicken sausages supplemented with up to 6% MP extract. The authors observed a decrease in the *L*^∗^ value from 68.5 in the control treatment to 59.4 in the treatment containing 6% MP extract. Additionally, the results are in agreement with the study reported by Mokhtar and Eldeep [53], where 0.3% MP extract was added to beef burgers.

On the other hand, treatment with pre-emulsified oils had greater effects on *L*^∗^ (*p* < 0.05) than treatment with saturated fat. An explanation for this could be that oil pre-emulsions have greater *L*^∗^ values than beef back fat. Oil globules in pre-emulsions are much smaller and reflect more light (more surface area) than larger animal fat globules. Rodríguez-Carpena, Morcuende, and Estévez [41] reported similar results to our study in which canola, avocado, and sunflower oil were added to beef burgers.

The addition of MP to the meat formulation increased (*p* < 0.05) the *a*^∗^ value. The high *a*^∗^ values, compared to those of treatments without MP, could be related to the protective and antioxidant effects of this ingredient. Abdel-Naeem et al. [54] obtained the same results when they added lemon, orange, and grapefruit peel powders to chicken burgers. On the other hand, oil pre-emulsion treatments had greater (*p* < 0.05) *a*^∗^ values than BF treatments. Choi et al. [55] reported similar results when backfat was substituted for grapeseed oil and rice bran fiber. These results can be attributed to the reaction between lipids and meat pigments, which affects the meat color [52]. During storage, the *a*^∗^ values decreased in the treatments without MP addition, possibly due to metmyoglobin formation; however, no significant differences were found in the beef patties with added MP (*p* > 0.05).

The addition of MP increased (*p* < 0.05) the *b*^∗^ value compared to treatments without MP. This may be due to the characteristic yellow color of the MP (*b*^∗^ = 36.45). With respect to the treatments containing pre-emulsified oils, the *b*^∗^ values increased (*p* < 0.05), possibly because oils and pre-emulsions had higher *b*^∗^ values than did BF (15.5, 12.0, and 9.0 for AO, SO, and BF, respectively; data obtained in our laboratory, not shown in this study), which affected the meat product color. Selani et al. [29] reported an increase in the *b*^∗^ value in beef patties supplemented with canola oil and pineapple peels due to the characteristic yellow color of the pineapple by-product (*b*^∗^ = 20.6). Regarding storage time, the *b*^∗^ values increased slightly (*p* < 0.05) in the evaluated treatments. Color evaluation is essential in meat products because it influences consumers' purchasing decisions.

Table 4 also shows the pH results of all of the evaluated treatments. Adding 1% MP significantly reduced the pH values in T4, T5, and T6 (*p* < 0.05). This decrease may be attributed to the acidic compounds found in MP, such as ascorbic acid, gallic acid, and ellagic acid [56, 57]. Selani et al. [29] reported similar results when pineapple peels were added to beef patties. The pH values remained unaltered with adding avocado and SO pre-emulsions (*p* > 0.05). However, the pH decreased as the storage period elapsed in treatments T1, T2, T3, and T4 (*p* < 0.05). Some researchers propose that bacteria, such as enterobacteria and lactic acid bacteria, can proliferate in meat during storage [58, 59]. Due to their acidic nature, these bacteria may contribute to the decrease in pH values.

On average, the pH decreased significantly in those treatments with avocado and SO pre-emulsions (*p* < 0.05). This decrease could be due to the slight acidity of the vegetable oils used in this study, as they had a lower pH (5.7) compared to the pH of BF (6.4) (data not shown). Serdaroğlu, Nacak, and Karabıyıkoğlu [60] reported similar results in chicken patties in which the saturated fat was replaced by a gelled emulsion prepared with olive oil. Thus, adding oils in the form of pre-emulsions can affect the pH values of reformulated meat products, with low pH values associated with the addition of vegetable oils.

#### 3.1.4. Lipid Oxidation During Cold Storage

**Figure 1:**
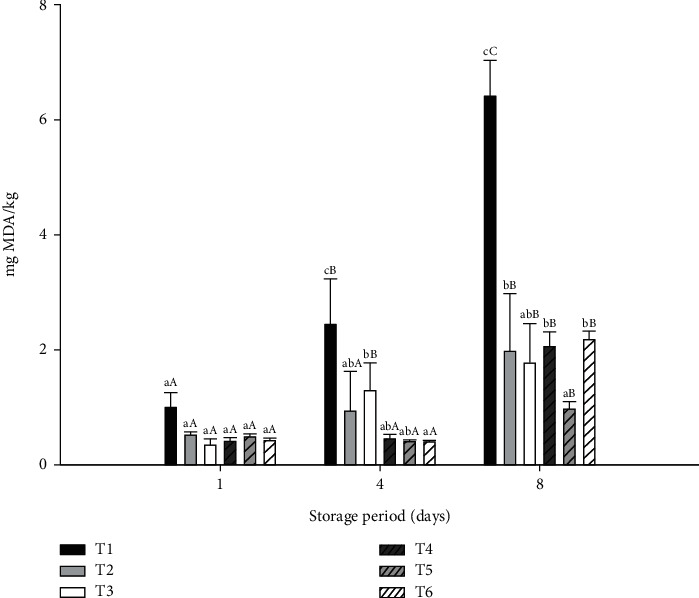
Lipid oxidation of cooked beef patties during storage, formulated with beef fat (BF), pre-emulsified avocado (AO) and safflower (SO) oils, and mango peel powder (MP). T1 = BF, T2 = AO, T3 = SO, T4 = BF + 1%MP, T5 = AO + 1%MP, and T6 = SO + 1%MP. ^A–C^Uppercase letters of the same treatment on different storage days are significantly different (*p* < 0.05); ^a–d^different lowercase letters on the same storage day are significantly different (*p* < 0.05).

The values of MDA obtained from the thiobarbituric acid test of beef patties supplemented with different types of fat and MP during storage are shown in Figure 1. The MDA values increased (*p* < 0.05) in all of the samples during the storage period. It is important to note that treatments T2, T3, T5, and T6, despite having the highest contents of unsaturated fatty acids, showed better oxidative stability during storage compared to treatments T1 and T4, with the highest values of SFAs. In agreement with our results, other authors have reported that meat products containing avocado and SOs have significantly lower levels of lipid oxidation products compared to the control treatments [41, 61]. Avocado has a substantial content of carotenoid and chlorophyll with proven antioxidant capacity [44, 62], while SO is rich in tocopherols, mainly *α*-tocopherol [45], and these properties could contribute to the oxidative stability in the treatments of our study containing vegetable oils. On the other hand, the addition of 1% MP decreased (*p* < 0.05) lipid oxidation compared to that in the treatments without MP during storage, possibly due to the TPC and antioxidant capacity of the MP [63]. Manzoor, Ahmad, and Yousuf [52] obtained similar results when they added MP extract to chicken sausages. A reduction in lipid oxidation in chicken cutlets was also observed by Bhat et al. [64] when 3% MP powder was incorporated into a meat formulation. Bioactive compounds such as gallic acid, protocateic acid, sinapic acid, caffeic acid, mangiferin, quercetin, and isoquercetin are found in MPs [65]. These bioactive compounds can help stabilize free radicals, slowing the oxidation process. These results show that MP could be used as a food ingredient in the preparation of meat products, such as beef patties, reducing lipid oxidation and preserving the quality of these products.

### 3.2. In Vitro Digestion Analysis

#### 3.2.1. FAAs

The lipid digestibility in foods can be estimated by measuring the in vitro release of FAAs after the simulated digestion process [66]. According to Hurt et al. [67], a higher FAA content in patties after in vitro digestion is indicative of increased total lipid digestibility. However, it is important to consider that the pro-oxidant conditions during digestion promote lipid oxidation, mainly in unsaturated fatty acids [14], resulting in a decrease in lipid digestibility. In this study, a by-product, MP, was proposed as a natural antioxidant to protect against lipid oxidation and increase the lipid digestibility of avocado and SOs added to the meat formulation.

**Figure 2:**
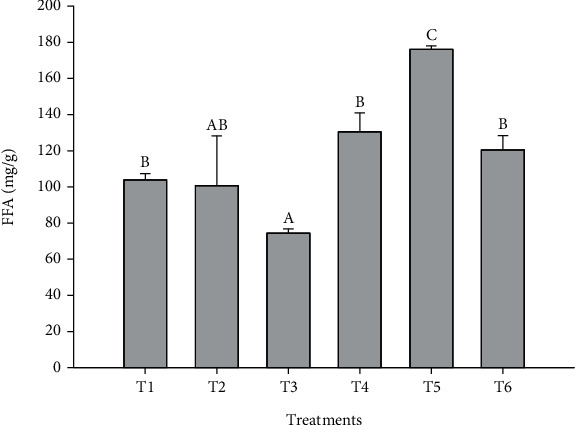
Free fatty acid (FFA) after in vitro digestion of cooked beef patties formulated with beef fat (BF), pre-emulsified avocado (AO) and safflower (SO) oils, and mango peel powder (MP). T1 = BF, T2 = AO, T3 = SO, T4 = BF + 1%MP, T5 = AO + 1%MP, and T6 = SO + 1%MP. Different letters between treatments are significantly different (*p* < 0.05).

Figure 2 shows the FFA content of cooked beef patties supplemented with different types of fat and with added MP after in vitro digestion. Among the treatments without MP addition, it was observed that the treatment with SO (T3) presented the lowest FFA content (*p* < 0.05). The lowest FFA values observed in T3 could be related to lipid oxidation since the digested samples also had the highest TBA values. This result is supported by studies showing that unsaturated lipids undergo oxidation catalyzed by meat iron under gastrointestinal conditions, resulting in high TBA values [68]. On the other hand, when comparing the treatments with MP, it was observed that the FFA values were greater (*p* < 0.05) in the treatment with added AO. The addition of 1% MP increased the FFA content after the in vitro digestion process, probably due to the antioxidant activity of MP. Preliminary studies in our laboratory showed that the content of phenolic compounds in the MP was 104.19 mg GAE/g, and the antioxidant activity was 113.2 and 211.1 mg TE/g for the equivalent antioxidant capacity of Trolox (TEAC) and DPPH tests, respectively. These values are considered high for the peel obtained from the Ataulfo cultivar compared to other varieties, such as the antioxidant capacity evaluated in the Haden MP (47 mg TE/g). The MP of the Ataulfo cultivar exhibits high antioxidant capacity, possibly due to its significant concentration of carotenoids and polyphenols, which include higher levels of lutein, mangiferin, catechin, and quercetin compared to peels from other mango varieties such as Kent, Manila, and Tommy Atkins [69, 70].

The presence of carotenoids and polyphenolic compounds in MP [63, 70] can contribute to the reduction of oxidation and loss of FFAs during digestion because they are involved in the elimination of free radicals, act as reducing agents, can chelate metals, and contribute to singlet oxygen quenching, among other processes [71].

Studies have shown that delaying the oxidation of food components during digestion can increase lipolysis [72, 73]. Nieva-Echevarria et al. [74] conducted a study that supported this hypothesis; they found that amino acids and peptides released during the in vitro digestion of ovalbumin and soy protein isolate exhibit antioxidant properties, which can affect the extent of lipid oxidation and promote the extension of lipolysis in sunflower and flaxseed oils.

The high values of FFAs in the treatments with MP obtained in the present investigation coincided with the results reported by Sobral et al. [73] in a study that evaluated the release of fatty acids in chicken meat patties during cooking and in vitro digestion when oregano or beer was added. According to the authors of this study, samples that were digested without added ingredients had the lowest content of PUFAs compared to those that were digested with added ingredients. This finding suggests that oregano or beer may help enhance the digestion of fatty acids or prevent lipid oxidation during digestion. MP is rich in phenolic compounds, phenolic acids, and carotenoids, among others, which can explain its ability to prevent lipid peroxidation. In this sense, cooking beef patties with the addition of 1% MP preserved the FFAs derived from the digestion of triglycerides.

#### 3.2.2. TPC and Antioxidant Capacity

**Figure 3:**
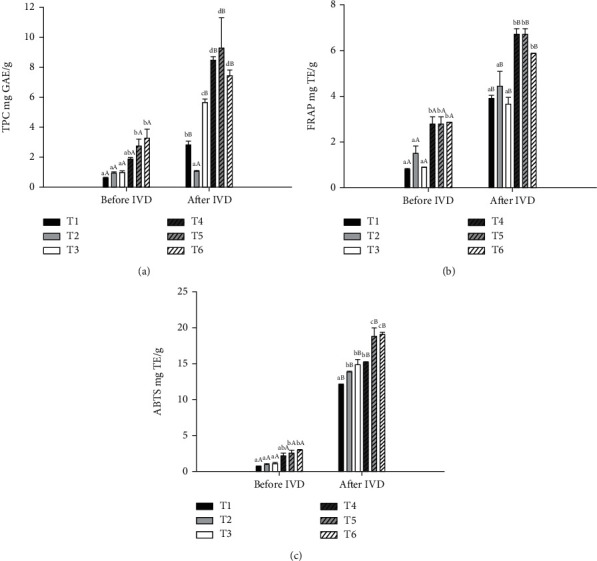
Total phenolic content (TPC) (a), FRAP (b), and ABTS (c) values before and after in vitro digestion (IVD) of cooked beef patties formulated with beef fat (BF), pre-emulsified avocado (AO) and safflower (SO) oils, and mango peel powder (MP). T1 = BF, T2 = AO, T3 = SO, T4 = BF + 1%MP, T5 = AO + 1%MP, and T6 = SO + 1%MP. ^A–B^Uppercase letters of the same treatment on different IVD steps are significantly different (*p* < 0.05); ^a–c^different lowercase letters in the same IVD steps are significantly different (*p* < 0.05).

The values obtained from the TPC of the beef patties substituted with different fat types and with added MP before and after in vitro digestion are shown in Figure 3(a). A comparison of the samples before digestion revealed that after the addition of MP, the TPC increased (*p* < 0.05); the same trend was observed after digestion, possibly due to the phenolic compounds in the MP, including gallic acid, mangiferin, ellagic acid, protocatechuic acid and quercetin [52]. The same results were found by Pešić et al. [75] in an in vitro digestion model of a food matrix based on meat and cereals enriched with grape extracts, where TPC increased when they were added. When comparing the TPC values before and after in vitro digestion, a higher content (*p* < 0.05) was found after digestion regardless of the addition of MP, possibly due to the release of compounds from the food matrix, which causes bond breakage by the action of digestive enzymes and conditions of the gastrointestinal tract when food is degraded [14]. The same effect was demonstrated by Trujillo-Mayol et al. [76], where the addition of avocado peel extract to beef and soy-based patties increased the TPC content before and after the digestion process.

When the treatments were compared after the digestion process, those with 1% MP had higher FT values (*p* < 0.05) than those without MP. The content of phenolic compounds in the MP increased, perhaps due to a more significant release of bioactive compounds from the food matrix caused by the breakdown of bonds derived from enzymatic hydrolysis. Furthermore, changing the pH from an acidic to an alkaline environment during intestinal digestion can improve the antioxidant capacity of phenols through the deprotonation of the hydroxyl groups present in their aromatic rings [77].

The FRAP test is a method used to measure the antioxidant capacity of food. The FRAP is based on the antioxidant capacity of reducing the Fe^3+^/ferricyanide complex to the ferrous form by donating an electron. After digestion, the antioxidant capacity of the cooked hamburgers was greater (*p* < 0.05) than that of the undigested samples (Figure 3(b)), reflecting greater bioaccessibility of bioactive compounds such as polyphenols, peptides, tocopherols, and carotenoids, possibly due to the release of bioactive compounds from the meat matrix. According to Biasi et al. [31], the bioaccessibility of phenolic compounds depends on the release of the matrix and its solubilization in the aqueous phase, as some can be complexed with food proteins or during digestion with enzymes and minerals. Increases in antioxidant capacity after in vitro digestion were also reported by Ansorena and Astiasaran [78] in pork patties containing rosemary and parsley, and by Torres-Martinez et al. [30] in cooked pork patties with added edible mushrooms. Structural changes in phenolic compounds attributed to enzymatic activity and variations in gastrointestinal pH could also contribute to the increase in antioxidant activity. As stated by Kim and Hur [79], digestive enzymes change the dietary polyphenolic compounds in pork patties containing tomato powder during in vitro digestion; this results in different structural forms with altered chemical properties and increased antioxidant capacity. The authors also suggest that the increase in antioxidant capacity is due to the release of phenolic compounds in pork patties during in vitro human digestion.

Furthermore, the iron reduction capacity increased with 1% MP in the meat formulation (*p* < 0.05), possibly due to the antioxidant capacity of MP to reduce ferric ions to their ferrous form, and this can be seen in the results obtained for TBA. Similar results were reported by Ryan et al. [80], who evaluated the addition of herbs to beef patties and reported a greater iron-reducing antioxidant potential before and after in vitro digestion. The antioxidant capacity of phenolic compounds is attributed to the number and location of the hydroxyl groups in their structure; however, the dietary fiber found in plant materials can prevent the absorption and stabilization of free radicals [81].

Figure 3(c) shows the TEAC in all of the evaluated treatments. The incorporation of MP significantly altered (*p* < 0.05) the ABTS radical inhibition activity in the undigested and digested samples. Compounds such as gallic acid, ellagic acid, mangiferin, and carotenoids contributed the most to its antioxidant activity.

After in vitro digestion, the antioxidant capacity of beef patties was greater (*p* < 0.05) than that of undigested samples, demonstrating that there was greater bioaccessibility of polyphenols and other compounds present in the raw materials after digestion. As mentioned earlier, some authors reported an increase in antioxidant capacity after the in vitro digestion of meat products containing natural antioxidants [31, 78, 79], mainly due to the release of polyphenol compounds from the food matrix and their further solubilization in digestive fluids. Additionally, digestive enzymes can transform phenolic compounds into different structural compounds with altered chemical properties and increased antioxidant activity [77]. The same results were found by Pešić et al. [75] using the method of simulated digestion of a food matrix based on meat and cereals enriched with grape extracts, who reported that the food matrix, digestive enzymes, and grape extracts contributed to the inhibitory activity of the ABTS radical. Therefore, an increase in total phenol content and antioxidant capacity could affect the results of the TBA determination, where the values could decrease due to delaying the oxidation process.

#### 3.2.3. Lipid Oxidation After In Vitro Digestion

**Figure 4:**
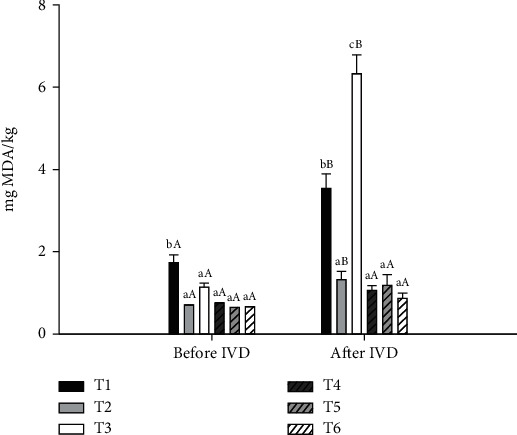
Lipid oxidation before and after in vitro digestion (IVD) of cooked beef patties formulated with beef fat (BF), pre-emulsified avocado (AO) and safflower (SO) oils, and mango peel powder (MP). T1 = BF, T2 = AO, T3 = SO, T4 = BF + 1%MP, T5 = AO + 1%MP, and T6 = SO + 1%MP. (A-B) Uppercase letters of the same treatment on different IVD steps are significantly different (*p* < 0.05); (a-c) different lowercase letters in the same IVD steps are significantly different (*p* < 0.05).

Lipid oxidation was measured by the TBA test. The values obtained from the TBA test of beef patties substituted with different types of fat and with added MP before and after in vitro digestion are shown in Figure 4. Before digestion, beef patties without added MP had a greater TBA content than those with 1% MP; this difference could be due to the total phenol content and the antioxidant capacity of MP (data not shown). However, compared with the saturated fat treatments, the pre-emulsified oil treatments decreased (*p* < 0.05) the TBA value, which may be due to the content of tocopherols in the oils. Previous studies in our laboratory showed that the alpha-tocopherol content in AO is greater than that in SO. The high alpha-tocopherol content in AO could explain why the treatments with AO pre-emulsions had lower TBA values. In general, TBA values increased (*p* < 0.05) after the digestion process, with values above 3 mg MDA/kg for the saturated fat treatments and the SO pre-emulsion without MP addition. In the digestive tract, food goes through pro-oxidant conditions such as low pH, enzymes, heme iron, and bile salts, causing an increase in lipid oxidation [14]. The main products derived from lipid oxidation after food lipid digestion are MDA, 4-HNE, and 4-HHE [82], all of which are implicated in oxidative damage in biological systems [15, 16]. Meat products with a high-fat content are more susceptible to lipid peroxidation due to the presence of the heme iron, which catalyzes the production of reactive oxygen species and the oxidation of unsaturated fatty acids [77], leading to increased lipid oxidation and generation of MDA, 4-HNE, and 4-HHE. These products derived from lipid oxidation could be implicated in chronic diseases such as cancer, hypertension, and atherosclerosis [83, 84]. Keller et al. [85] demonstrated that the administration of heme iron in a diet supplemented with linoleic acid-rich SO in rats induces the production of 4-HNE. The authors suggested that the formation of HNE-protein adducts in heart, liver, and skeletal muscle tissues could be implicated in the development of chronic diseases.

In the treatments with 1% MP, there was also a significant reduction (*p* < 0.05) in the TBA levels compared with those in the beef patties without MP addition. The total phenol content and the antioxidant capacity of the MPs contributed to the reduction in lipid oxidation. MP contains bioactive compounds such as gallic acid, protocateic acid, sinapic acid, caffeic acid, and mangiferin, which can stabilize free radicals [65]. Polyphenols can react directly with RO^•^ and ROO^•^, decreasing the propagation and inhibiting the chain reaction of free radicals. Studies carried out by Van Hecke et al. [86] showed that the addition of spices and herbs to high-fat beef reduced the formation of MDA during in vitro digestion. Han et al. [87] also reported a significant inhibition of MDA after the in vitro digestion of high-fat beef containing olive polyphenols, especially when it reached 600 mg/kg in the meat formulation. Lavado and Cava [88] reported similar results in dry sausage with added pomegranate peel extract (PPE). These authors reported a decrease in MDA, 4-HNE, and lipid hydroperoxides after the in vitro digestion of meat samples with 1% PPE compared with meat samples without PPE. During the cooking and digestion process, the food matrix may be softened, which causes a rupture of the cell walls, increasing the release of free phenolic compounds and those bound to the fiber [89], resulting in a decrease in MDA and other lipid oxidation products. In our study, 1% MP in beef patties reduced lipid oxidation before and after in vitro digestion.

## 4. Conclusion

The results of this work highlight the substitution of vegetable oils and the addition of MP as alternatives to improve the quality parameters and oxidative stability of meat products without affecting their physicochemical properties. By-products derived from fruit processing, such as MP, can be used as ingredients in meat formulations susceptible to oxidation, such as those in which saturated fat has been replaced by vegetable oils, preserving the meat's quality, and reducing lipid oxidation during storage and digestion. The results of this research may be useful for food processors interested in developing new meat products.

## Figures and Tables

**Table 1:** Chemical composition of beef patties formulated with beef fat (BF), pre-emulsified avocado (AO) and safflower (SO) oils, and mango peel powder (MP).

**Chemical composition**	**Treatments**						**SEM**
	**T1**	**T2**	**T3**	**T4**	**T5**	**T6**	
Moisture	55.72	54.10	53.36	53.88	54.13	54.16	0.665
Fat	11.49	13.49	13.66	13.21	12.38	11.49	0.475
Protein	25.01	26.60	26.88	25.95	26.81	27.51	0.355
Ash	5.49	4.85	4.67	4.87	4.45	4.88	0.190

*Note:*T1 = BF, T2 = AO, T3 = SO, T4 = BF + 1%MP, T5 = AO + 1%MP, and T6 = SO + 1%MP.

Abbreviation: SEM = standard error of the means.

**Table 2:** Fatty acid profile of beef fat, avocado oil, and safflower oil used for the preparation of beef patties.

**Fatty acid** ^a^	**Beef fat**	**Avocado oil**	**Safflower oil**
C14:0	4.90	0.05	0.04
C15:0	ND	0.05	0.01
C16:0	22.10	8.31	4.59
C17:0	1.47	0.06	0.03
C18:0	21.03	2.00	2.93
C20:0	0.54	0.48	0.32
C22:0	ND	0.25	0.85
C14:1	ND	ND	ND
C16:1	2.7	0.98	0.11
C17:1	ND	0.06	0.04
C18:1 *trans*	ND	ND	ND
C18:1 *cis*	45.35	49.60	75.04
C20:1	0.19	0.89	0.29
C18:2 *trans*	ND	ND	ND
C18:2 *cis*	1.58	31.11	15.12
C18:3 *n3*	0.14	6.17	0.34
C20:3 *n6*	0.19	ND	ND
C20:4 *n6*	0.62	ND	ND
∑ SFA	49.31	11.20	8.76
∑ MUFA	48.21	51.58	75.48
∑ PUFA	2.48	37.28	15.45

Abbreviations: MUFA = monounsaturated fatty acid, ND = not detected, PUFA = polyunsaturated fatty acid, SFA = saturated fatty acid.

^a^Values express as percentage of the total of fatty acids identified.

**Table 3:** Fatty acid profile of cooked beef patties formulated with beef fat (BF), pre-emulsified avocado (AO) and safflower (SO) oils, and mango peel powder (MP).

**Fatty acid** ^**∗**^	**Treatments**						**SEM**
	**T1**	**T2**	**T3**	**T4**	**T5**	**T6**	
C14:0	3.07^b^	1.42ª	1.44ª	3.35^b^	1.40ª	1.37ª	0.38
C15:0	0.44^b^	0.21ª	0.21ª	0.49^b^	0.21ª	0.20ª	0.05
C16:0	25.07^b^	15.89ª	13.40ª	27.23^b^	15.74ª	14.32ª	2.43
C17:0	1.26^b^	0.59ª	0.57ª	1.36^b^	0.59ª	0.57ª	0.15
C18:0	16.61^b^	8.59ª	8.47ª	18.54^b^	8.60ª	8.80ª	1.90
C20:0	0.13ª	0.33^c^	0.24^b^	0.14ª	0.31^c^	0.25^b^	0.03
C22:0	ND	0.18ª	0.45^b^	ND	0.18ª	0.46^b^	0.07

C14:1	0.68^b^	0.34ª	0.41ª	0.71^b^	0.33ª	0.34ª	0.07
C16:1	3.21^b^	2.02ª	1.76ª	3.41^b^	2.01ª	1.65ª	0.31
C17:1	0.83^b^	0.42ª	0.45ª	0.87^b^	0.42ª	0.42ª	0.08
C18:1 *trans*	3.10^b^	1.47ª	1.69ª	3.46^b^	1.55ª	1.59ª	0.36
C18:1 *cis*	39.29ª	45.54^b^	57.59^c^	33.86ª	44.92^b^	56.68^c^	3.83
C20:1	0.20ª	0.58^c^	0.30^b^	0.19ª	0.54^c^	0.29^b^	0.05

C18:2 *trans*	0.33^b^	0.17ª	0.21ª	0.32^b^	0.15ª	0.16ª	0.03
C18:2 *cis*	4.27ª	17.56^c^	11.54^b^	4.49ª	16.97^c^	11.36^b^	2.35
C18:3 *n3*	0.23ª	3.48^c^	0.54^b^	0.32^ab^	3.31^c^	0.55^b^	0.63
C20:3 *n6*	0.26ª	0.20ª	0.13ª	0.28ª	0.19ª	0.17ª	0.05
C20:4 *n6*	0.93ª	0.74ª	0.58ª	0.99ª	0.73ª	0.64ª	0.06

∑ SFA	46.59^b^	27.22ª	24.79^a^	51.10^b^	26.93^a^	25.97^a^	5.67
∑ MUFA	47.30^b^	50.38^b^	62.21^c^	42.50^a^	49.59^b^	60.97^c^	3.10
∑ PUFA	6.11^b^	22.41^c^	13.00^b^	6.40ª	21.36^c^	12.88^b^	2.86

*Note:*T1 = BF, T2 = AO, T3 = SO, T4 = BF + 1%MP, T5 = AO + 1%MP, and T6 = SO + 1%MP. Different letters (a–c) within the same row are significantly different (*p* < 0.05).

Abbreviations: ND = not detected; MUFA = monounsaturated fatty acid; PUFA = polyunsaturated fatty acid; SFA = saturated fatty acid; SEM = standard error of the means.

^*^Values expressed as a percentage of the total of fatty acids identified.

**Table 4:** Instrumental color and pH of cooked beef patties formulated with beef fat (BF), pre-emulsified avocado (AO), safflower (SO) oils, and mango peel powder (MP).

**Parameter**	**Treatments**	**Storage period (days)**			**SEM**
		**1**	**4**	**8**	
*L* ^∗^	T1	51.33^abA^	53.02^bcA^	53.92^cA^	0.72
	T2	49.86^abA^	54.81^cAB^	56.10^cB^	1.01
	T3	53.28^bA^	53.83^cA^	53.87^cA^	0.43
	T4	49.34^abB^	42.97^aA^	47.66^aAB^	1.03
	T5	46.89^aA^	50.91^bcB^	50.72^abAB^	0.84
	T6	49.75^abA^	48.18^bA^	51.39^abcA^	0.63

*a* ^∗^	T1	6.24^aB^	4.70^aA^	4.66^aA^	0.22
	T2	6.71^aAB^	5.87^bB^	5.36^aA^	0.16
	T3	7.03^aB^	6.38b^bAB^	5.55^aA^	0.14
	T4	7.29^bA^	7.80^cA^	7.82^bA^	0.11
	T5	8.21^cA^	8.25^cA^	8.09^bA^	0.00
	T6	8.05^bcA^	8.23^cA^	8.34^bA^	0.00

*b* ^∗^	T1	12.64^aA^	13.19^aA^	13.18^aA^	0.25
	T2	13.65^abA^	13.86^abA^	14.48^abA^	0.15
	T3	13.44^abA^	13.78^abAB^	14.99^bB^	0.26
	T4	14.62^bcA^	16.88^cB^	15.78^bA^	0.34
	T5	15.94^cA^	14.98^bA^	14.54^abA^	0.26
	T6	14.60^bcA^	13.75^aA^	15.29^bA^	0.23

pH	T1	6.38^cB^	6.24^bA^	6.21^bA^	0.02
	T2	6.30^bcB^	6.21^bA^	6.20^bA^	0.02
	T3	6.37^cC^	6.21^bB^	5.97^aA^	0.02
	T4	6.19^bA^	6.16^bA^	6.11^bA^	0.02
	T5	6.03^aA^	5.93^aA^	5.90^aA^	0.02
	T6	6.09^abB^	6.00^aAB^	5.88^aA^	0.02

*Note:*T1 = BF, T2 = AO, T3 = SO, T4 = BF + 1%MP, T5 = AO + 1%MP, and T6 = SO + 1%MP. Different uppercase letters (A–C) within the same row are significantly different (*p* < 0.05); different lowercase letters (a–c) in the same column are significantly different (*p* < 0.05).

## Data Availability

All data are provided in this manuscript.
